# Canstatin induces apoptosis in gastric cancer xenograft growth in mice through the mitochondrial apoptotic pathway

**DOI:** 10.1042/BSR20140012

**Published:** 2014-04-25

**Authors:** Ya-Nan Xing, Peng Deng, Hui-Mian Xu

**Affiliations:** Department of Surgical Oncology, First Affiliated Hospital of China Medical University, Shenyang 110001, China

**Keywords:** canstatin, chick chorioallantoic membrane, gastric cancer, mitochondrial apoptotic pathway, survival rate, CAM, chorioallantoic membrane, DAB, 3,3′-diaminobenzidine tetrahydrochloride, VEGF, vascular endothelial growth factor, HRP, horseradish peroxidase

## Abstract

Canstatin, the non-collagenous domain of collagen type IV α-chains, belongs to a series of collagen-derived angiogenic inhibitors. In this study, the inhibitory effect of recombinant canstatin on tumour growth was investigated using a gastric cancer xenograft model. The volume and weight of tumours in mice treated with canstatin were lower than that in mice treated with PBS. Accordingly, the survival rate of these mice was significantly higher than that of mice bearing tumours treated with PBS. Moreover, valuable insight into the mechanisms mediated by canstatin was obtained.

## INTRODUCTION

Gastric cancer is the second most common cause of cancer deaths worldwide. A total of 989600 new gastric cancer cases and 738000 deaths are estimated to have occurred in 2008 [1]. About 60% of new cases of gastric cancer occur in eastern Asia [2], especially in China. In recent years, molecular biology methods for treatment in gastric cancer have been given great concern. Some potential molecular targets for therapy in gastric cancer have been reported in previous studies, such as EGFR (epidermal growth factor receptor) [3], VEGF (vascular endothelial growth factor) [4] and RON (recepteur d’origine nantais) [5].

Angiogenesis is crucial for the growth and metastasis of solid tumours [6]. The type IV collagen is a crucial component of vascular basement membrane, which consists of six genetically distinct α-chains: α1 (IV), α2 (IV), α3 (IV), α4 (IV), α5 (IV) and α6 (IV) [7,8]. Canstatin is the 24 kDa non-collagenous (NC1) domain of the α2 chain of type IV collagen [9]. Previous studies have shown that canstatin could inhibit endothelial cell migration and tube formation [9–11]. In addition, canstatin inhibits tumour growth in mouse models [10] and is a potent inhibitor of angiogenesis with a distinct antitumour activity [12].

We further investigated the anti-angiogenesis and antitumour activity of canstatin and our results showed that it can inhibit the neovascularization of chick CAM (chorioallantoic membrane) and suppress the growth of SGC-7901 in a xenograft model of nude mice through mitochondrial apoptotic pathway. These findings suggest that canstatin could suppress the proliferation of gastric cancer cell and vascular endothelial cell. This finding may be useful in improving the clinical effectiveness of canstatin for the treatment of gastric cancer.

## MATERIALS AND METHODS

### Cell lines and culture

Human gastric cancer cell, SGC-7901, was obtained from the American Type Culture Collection. The cell lines were grown in the RPMI 1640 medium (Hyclone) supplemented with 10% (v/v) FBS and antibiotics (100 units/ml penicillin and 100 μg/ml streptomycin) and maintained in a humidified cell incubator with 5% (v/v) CO_2_ at 37°C.

### Chick CAM angiogenesis assay

Angiogenesis was induced on CAMs of 6-day-old chick embryos by placing a filter disc containing VEGF (approximately 0.1 μg/1 μl) on the CAMs as described previously [13]. The embryos were treated with recombinant canstatin (restored in our laboratory) [14]. The embryos were incubated for a total of 3 days at which time the filter disc was removed and surrounding CAM tissues were fixed, excised and photographed. The image was acquired with a digital scanner (Epson). Five square regions were selected from the digitized image, binarized to black and white, skeletonized and the total length of the vessels in each region was measured using the NIH Image 1.61 program [15]. These experiments were completed performed three times with five embryos per condition. The concentrations of high recombinant canstatin (suppressed angiogenesis) and low recombinant canstatin (not suppressed angiogenesis) were determined and used in the following studies.

### Animal experiments

All experiments with animals were performed according to the guidelines of China Medical University Ethical Committee. Human gastric cancer xenografts were established in 4–6-week-old nude mice (Experimental Animal Center of Academy of Sciences, Shanghai, China) by subcutaneous inoculation of 3×10^7^ SGC-7901 cells into the axilla of each mouse. After the tumour diameters reached 3–5 mm, the mice were divided randomly into three groups (PBS, high canstatin and low canstatin) and received a 1-ml intratumoural injection of PBS, high canstatin (10 μg/100 μl) or low canstatin (5 μg/100 μl). PBS, high canstatin or low canstatin were injected into five different positions of tumour. Each point received 200 μl injections. Tumour growth was then monitored for 28 or 35 days. Every 7 days until the end of the experiment, one mouse from each group was randomly selected to be anesthetized, photographed and killed. The tissues recovered were subjected to further analysis. For each tumour, measurements were made using calipers, and tumour volumes were calculated as follows: length×width^2^×0.52 [16].

### Survival curves

Additional mice (*n*=90) were used to establish xenografts to obtain survival curves. Mice with xenografted tumours (as described above) that reached 3–5 mm in diameter were divided into three treatment groups (*n*=30 for each). Survival was monitored until the experiments were terminated due to the heavy tumour burden.

### Immunostaining

For immunohistochemical staining of CD31 or caspase 3, endogenous peroxidase activity was blocked in 4-μm tumour sections with 3% (v/v) hydrogen peroxide for 30 min. Antigen retrieval was performed in citrate buffer (10 mM, pH 6) for 30 min at 95°C in a pressure cooker. CD31 antibody (Sigma) or caspase 3 antibody (Santa Cruz) was incubated with sections at 1:500 overnight at 4°C. Sections were then incubated with a biotinylated secondary antibody for 1 h at room temperature, followed by incubation with a streptavidin HRP (horseradish peroxidase) complex (Beyotime) for 60 min at room temperature. Bound antibody was visualized with DAB [3,3′-diaminobenzidine tetrahydrochloride (diaminobenzidine), Beyotime). Sections were also counterstained with haematoxylin (Beyotime).

### Quantification of intratumoural microvessels

Regions of highest vessel density were located at low magnification (40×), then the number of vessels present were counted at 200× magnification. Three high magnification fields were counted for each tumour section and the mean microvessel density value was recorded for each. Any individual endothelial cells, or endothelial cell cluster, that was clearly separated from adjacent microvessels was counted as a single microvessel.

### Western blot analysis

Cells and tissues were collected, washed twice with PBS, lysed on ice for 30 min in 100 μl lysis buffer and then centrifuged at 13 000***g*** for 15 min. The supernatants were collected from the lysates and the protein concentration was determined. Aliquots of the lysates (15 μg of protein) were boiled for 5 min and electrophoresed using a SDS/10% PAGE. The blots in the gels were transferred onto nitrocellulose membranes (Bio-Rad), which were then incubated with primary antibodies. Bax (sc-7480, 1:200), Bcl-xL (sc-8392, 1:200), Bcl-2 (sc-783, 1:200), phospho-Bcl-2 (Ser^87^) (sc-16323, 1:200) and β-actin (sc-103656, 1:1000) antibodies were purchased from Santa Cruz Biotechnology. The nitrocellulose membranes were further incubated with secondary immunoglobulin-G-HRP conjugates. Immunostaining was detected using an ECL® (enhanced chemiluminescence) system (Amersham Biosciences). Densitometric quantification of the target proteins was performed with a β-actin control using Scion Image (Version 4.0.3.2, Scion Corporation) for Windows.

### Statistical analysis

Values are presented as mean and SD for these experiments (mean±S.D.). Statistical significance was calculated with a Mann–Whitney–Wilcoxon statistical test. *P*-values <0.05 were considered significant. Each result in this work is representative of at least three separate experiments.

## RESULTS

### Effect of canstatin on *in vivo* angiogenesis

**Figure 1:**
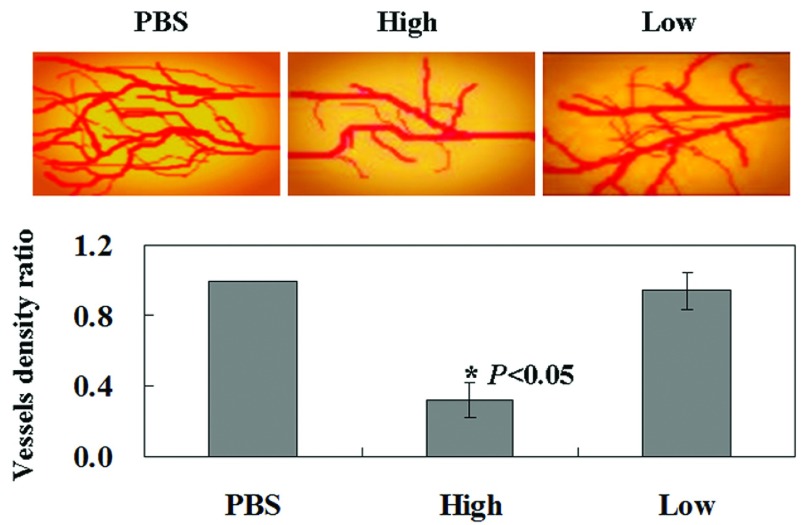
Canstatin potentially inhibits VEGF-induced angiogenesis *in vivo* in the chick embryo model Embryos were treated with high canstatin (10 μg/embryo) or low canstatin (5 μg/embryo), the negative control treated with PBS.

In order to evaluate the inhibitory effect of canstatin on *in vivo* angiogenesis, we tested this possibility on VEGF-induced angiogenesis in the chick CAM. The results showed canstatin inhibits neovascularization with the chick CAM model, and this inhibition was dependent on the concentration (Figure 1). In addition, the administration of high canstatin (10 μg/embryo) resulted in potential inhibition of neovascularization with the chick CAM model, in contrast, the treatment with low canstatin (5 μg/embryo) showed no effect on neovascularization (Figure 1).

### Effect of canstatin on the growth of tumour in mouse xenograft model

**Figure 2:**
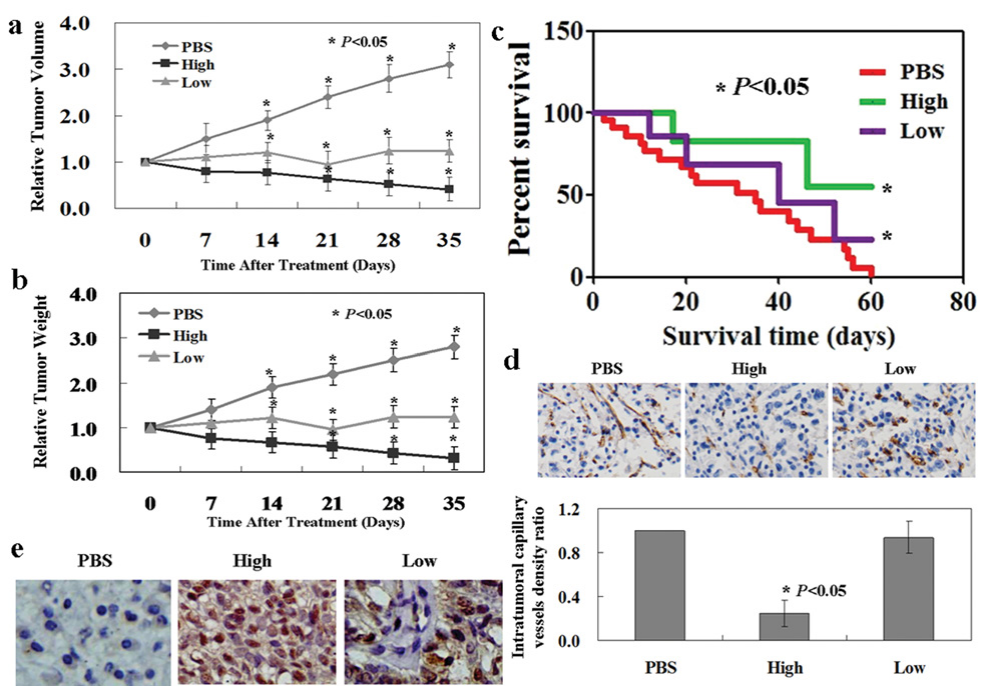
Canstatin suppresses SGC-7901 cell tumour growth *in vivo* (**a**, **b**) Tumour xenografts were established in nude mice with subcutaneous injections of SGC-7901 cells (3×10^7^ in 200 μl). After tumours reached diameters of 3–5 mm, tumours were directly injected with PBS, high canstatin and low canstatin. Tumour volume and tumour weights measured for each tumour group on days 7, 14, 21, 28 and 35. (**c**) Kaplan-Meier survival curves for mice bearing SGC-7901 treated with PBS (red), high canstatin (green) or low canstatin (purple). Each experiment was performed in triplicate. (**d**) Immunohistochemical staining of tumour vessel endothelial cells using an anti-CD31 antibody. (**e**) Immunohistochemical staining of apoptotic cancer cells using an anti-caspase 3 antibody. Bound antibody is detected with DAB and appears brown.

We next examined the effect of canstatin on established primary mouse tumour model. Models were established with the subcutaneous injection of treated SGC-7901 cells (3×10^7^ in 200 μl) into the axilla of 4–6-week-old nude mice. On day 35, a significant inhibition of tumour volume was observed for tumour cells treated with canstatin. For example, compared with the cells treated with PBS (504±43 mm^3^) and low canstatin (286±28 mm^3^), high canstatin-treated cells had a tumour volume of 114±32 mm^3^ (Figure 2A, *P*<0.05). Correspondingly, the weights of PBS, low canstatin and high canstatin-treated tumours followed the same order (e.g., 491±24, 312±27 and 121±25 mg, respectively) (Figure 2B, *P*<0.05). In addition, the survival rate of mice with tumours treated with high canstatin or low canstatin was significantly improved. For example, while mice in the PBS group began to die on day 2, mice in the high canstatin-treated group did not die until day 24 or low canstatin group did not die until day 9. Moreover, by the end of the experiment, only three mice died in the high canstatin group, while all of the mice in the PBS groups had died (Figure 2C, *P*<0.05). To address the possibility that canstatin inhibited tumour growth by suppressing angiogenesis, immunohistochemical staining of CD31 was performed for tumour sections. The blood vessel density of tumours pretreated with low canstatin was not significantly reduced compared with that of the untreated group (Figure 2D, *P*>0.05). We also found that the apoptotic cancer cell in the high canstatin group is higher than the low canstatin group or the PBS group, and the apoptotic cancer cell in the low canstatin group is higher than the PBS group (Figure 2E).

### The mechanism(s) of canstatin in mouse xenograft model

**Figure 3:**
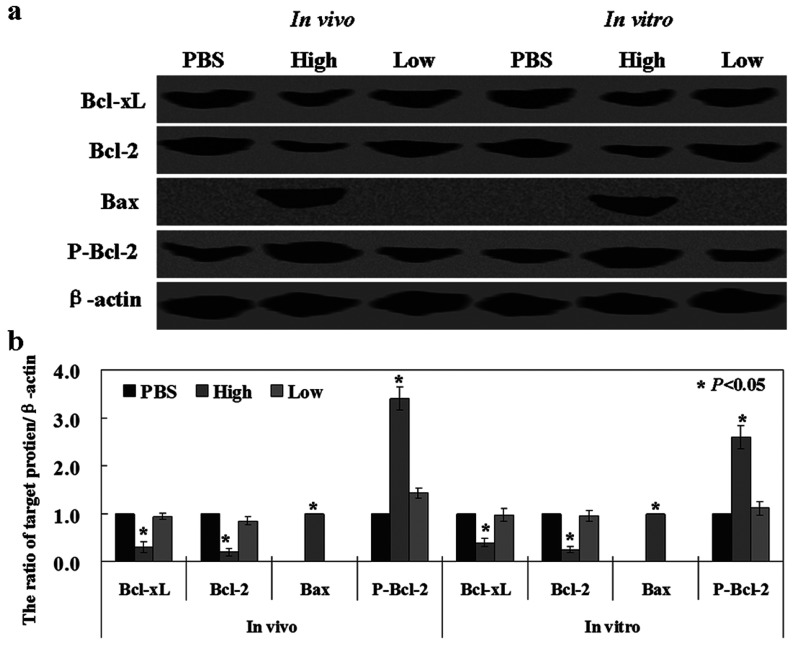
Canstatin exhibits anti-tumour activity via mitochondrial apoptotic pathway (**A**) Levels of Bcl-xl, Bcl-2, Bax and phosphorylated forms of Bcl-2 were detected in Western blots of tissue or cell lysates. β-actin was used as an internal control. (**B**) Densitometric quantification of the target proteins was performed with a β-actin control using Scion Image. The histogram shows the results has statistical significant (*P*<0.05).

In order to identify the mechanism of canstatin, we detected the protein expression of Bax, Bcl-2 and Bcl-xl, which are members of the Bcl-2 family, using Western blot analysis. We found a decrease in Bcl-2 and Bcl-xl protein, and an increase in Bax protein levels both in the tumour tissues of the mice and tumour cells treated with canstatin (Figure 3, *P*<0.05). We noted that the phosphorylation of Bcl-2 was increased in the tumour tissues and cells treated with high concentration of canstatin (Figure 3, *P*<0.05).

## DISCUSSION

Previous studies have shown that tumstatin inhibits proliferation of endothelial cells *in vitro* and suppress tumour growth *in vivo* [16–19]. To our knowledge, no previous studies showed the roles of canstatin in cancer cells *in vivo*. Our previous study showed that canstatin could induce apoptosis and G_1_ arrest in colorectal cancer cells, HCT-15 and HCT-116 [14]. It is interesting to investigate whether canstatin could inhibit tumour itself *in vivo*.

Initially, we investigated the *in vivo* antiangiogenic properties of canstatin by using chick CAM angiogenesis assay. We confirmed canstatin could inhibit proliferation of endothelial cells and chose a low concentration of canstatin that could not induce apoptosis in endothelial cells.

Next, we attempted to delineate antitumour activities of canstatin on gastric cancer in a mouse xenograft model. In order to avoid the antiangiogenic properties of canstatin, we used the low concentration of canstatin to treat the xenograft model. By using the xenograft model, we observed that a decrease in tumour size and tumour weight was not consistent with a decrease in angiogenesis. However, tumour size and tumour weight of the mice treated with the high concentration of canstatin were significantly lower than that of the mice treated with the low concentration of canstatin. These results indicated that canstatin could suppress both endothelial cells and cancer cells.

Furthermore, we confirmed the possible mechanism of canstatin in SGC-7901. According to a previous study, Bcl-2 and Bcl-xl are two anti-apoptotic proteins, and Bax is a pro-apoptotic protein [20]. The balance of pro-apoptotic and anti-apoptotic members controls the sensitivity of cells to apoptosis [21]. In line with the earlier findings, we observed reductions in both the Bcl-2 and Bcl-xl expression in cells treated with canstatin. Consistent with Xia’ results [22], we also found that p-Bcl-2, an inactivated form of Bcl-2, was increased significantly. Panka et al. [23] found that canstatin-induced apoptosis in HUVEC (human umbilical vein endothelial cells) is associated with phosphatidylinositol 3-kinase/Akt (protein kinase B) inhibition. In this study, our results suggested that canstatin promotes apoptosis both in endothelial cells and cancer cells via mitochondrial apoptotic pathway that included a reduction of Bcl-2 and Bcl-xl proteins, and an induction in Bax protein. These results indicated that the mechanisms of canstatin are involved in many signalling pathways.

In summary, the principal findings of our study are that: (1) the cytotoxicity of canstatin in gastric cancer cell and (2) canstatin-induced apoptosis in SGC-7901 cells via mitochondrial apoptotic pathway.
